# Discussion on the Treatment Strategy for Stage ⅡA1 Cervical Cancer (FIGO 2018)

**DOI:** 10.3389/fonc.2022.800049

**Published:** 2022-04-14

**Authors:** Xiaolin Chen, Wentong Liang, Hui Duan, Minling Wu, Xuemei Zhan, Encheng Dai, Qiubo Lv, Qinghuang Xie, Ruilei Liu, Yan Xu, Xiaonong Bin, Jinghe Lang, Ping Liu, Chunlin Chen

**Affiliations:** ^1^ Department of Obstetrics and Gynecology, Nanfang Hospital, Southern Medical University, Guangzhou, China; ^2^ Department of Obstetrics and Gynecology, Guizhou Provincial People’s Hospital, Guizhou, China; ^3^ Department of Obstetrics and Gynecology, Jiangmen Central Hospital, Jiangmen, China; ^4^ Department of Obstetrics and Gynecology, Linyi People’s Hospital, Linyi, China; ^5^ Department of Obstetrics and Gynecology, Beijing Hospital, Beijing, China; ^6^ Department of Gynecology, Foshan Maternal and Child Health Hospital, Foshan, China; ^7^ Department of Obstetrics and Gynecology, Pan Yu Central Hospital, Guangzhou, China; ^8^ Department of Epidemiology, College of Public Health, Guangzhou Medical University, Guangzhou, China; ^9^ Department of Obstetrics and Gynecology, Peking Union Medical College Hospital, Peking Union Medical College, Beijing, China

**Keywords:** cervical cancer, stage ⅡA1, overall survival, disease-free survival, treatment strategies

## Abstract

**Objective:**

This study aimed to explore the best treatment strategy for International Federation of Gynecology and Obstetrics (FIGO) 2018 stage IIA1 cervical cancer patients by comparing the survival outcomes of two treatment methods: abdominal radical hysterectomy (ARH) with standard postoperative therapy and radio-chemotherapy (R-CT).

**Methods:**

Patients with FIGO2018 stage IIA1 cervical cancer who underwent ARH or received R-CT were screened from the clinical diagnosis and treatment for cervical cancer in China (Four C) database. The recurrence cases between the two groups were analyzed. The 5-year overall survival (OS) and disease-free survival (DFS) of patients diagnosed with stage IIA1 cervical cancer in 47 hospitals in mainland China between 2004 and 2018 were compared by using propensity score matching (PSM).

**Results:**

A total of 724 patients met the inclusion criteria. In the total study population, The R-CT group had higher recurrence (22.8% for the R-CT group and 11.2% for the ARH group, *P*<0.001) rates compared to the ARH group.The 5-year OS and DFS of the ARH group (n=658) were significantly higher than those of the R-CT group (n=66) (OS: 85.9% *vs*. 71.2%, *P*=0.009; DFS: 79.2%*vs*. 70.5%, *P*=0.027). R-CT was associated with worse 5-year OS (HR=3.19, 95% CI: 1.592-6.956, *P*=0.001) and DFS (HR=2.089, 95% CI: 1.194-3.656, *P*=0.01). After 1:2 PSM, the 5-year OS and DFS of the ARH group (n=126) were significantly higher than those of the R-CT group (n=64) (OS:88.9% *vs.* 70.1%, *P*=0.04; DFS:82.8% *vs.* 69.8%, *P*=0.019). R-CT was still associated with worse 5-year OS (HR=2.391, 95% CI: 1.051-5.633, *P*=0.046) and DFS (HR=2.6, 95% CI: 1.25-5.409, *P*=0.011).

**Conclusion:**

Our study demonstrated that for stage FIGO2018 stage IIA1 cervical cancer patients, ARH offers better oncological outcomes than R-CT.

## Introduction

All cases of lymph node metastasis were classified as newly established International Federation of Gynecology and Obstetrics (FIGO) 2018 stage IIIC (1, 2), so FIGO2018 stage IIA1 was redefined as follows: cancerous lesion with invasion that exceeded the cervix but did not reach 1/3 of the vagina or the pelvic wall; maximum tumour diameter of the cancerous stove ≤4 cm; and no metastasis to the pelvic lymph nodes. However, for the treatment of FIGO2018 stage IIA1 cervical cancer patients, surgical treatment or curative chemotherapy could be chosen (3). Since FIGO2018 stage IIA1 cervical cancer no longer includes lymph node metastasis, it was worth exploring whether the optimal treatment strategy has also changed. Based on the above questions, this study was intended to screen FIGO2018 stage IIA1 cervical cancer patients from the clinical diagnosis and treatment for cervical cancer in China (Four C) database. We compared the oncological outcomes of abdominal radical hysterectomy (ARH) with standard postoperative therapy and radio-chemotherapy (R-CT) to explore the best treatment strategies for patients with FIGO2018 stage IIA1 cervical cancer.

## Materials and Methods

### Data Source

We systematically collected clinical data on 63,926 patients with cervical cancer at each stage in 47 hospitals in mainland China between 2004 and 2018 and tracked patients’ long-term oncological outcomes. This study was a multi-centre retrospective cohort study approved by the Southern Hospital Ethics Committee of Southern Medical University (Ethics number NFEC-2017-135). The clinical trial identifier is CHiCTR1800017778 (International Clinical Trials Registry Platform Search Port, http://apps.who.int/trialsearch/). Data collection and follow-up were carried out by gynecologists who had received unified training. After the completion of data entry, two independent gynecologists double-entered the data and checked the information to ensure the accuracy of the data. Relevant data collection and database construction procedures can be found in papers published by our team (4–8). Among them, the cases in this database collected before 2009 were staged by FIGO 1994, and the cases collected after 2009 were staged by FIGO2009. All the cases were re-corrected according to the FIGO2018 cervical cancer staging system after being entered into the database.

### Inclusion and Exclusion Criteria

In this study, the inclusion and exclusion criteria were as follows:

Inclusion criteria for the ARH with postoperative standard therapy group (ARH group): (1) age ≥18 years old; (2) cervical biopsy pathology diagnosis of cervical cancer; (3) postoperative histological types: squamous cell carcinoma, adenocarcinoma, and adenosquamous cell carcinoma; (4) clinical stage IIA1 (2018) disease; (5) no neoadjuvant chemotherapy or radiotherapy; (6) surgical approach: abdominal; (7) operation type: Querleu–Morrow type C hysterectomy + pelvic lymph node excision ± abdominal para-aortic lymph node excision/biopsy; (8) complete postoperative pathological report and information on the lymph node status (negative pelvic lymph node and para-aortic lymph node metastases); (9) postoperative adjuvant treatment: standard; and (10) attending follow-up visits.

Inclusion criteria for the R-CT group: (1) age ≥18 years old; (2) cervical biopsy pathology diagnosis of cervical cancer; (3) histological types: squamous cell carcinoma, adenocarcinoma, and adenosquamous cell carcinoma; (4) FIGO2018 stage: stage IIA1; (5) pre-treatment MRI or CT examination and description of the lymph node status (negative for pelvic lymph node and para-aortic lymph node metastases); (6) initial treatment: R-CT, treatment including pelvic external beam radiotherapy+vaginal brachytherapy, with a radiotherapy dose≥85 Gy, including concurrent platinum-containing chemotherapy; and (7) attending follow-up visits.

The exclusion criteria were as follows: (1) did not meet the abovementioned admission criteria; and (2) pregnancy combined with cervical cancer, residual cancer, or another malignancy.

### Observation Indicators

The observation endpoints were overall survival (OS) and disease-free survival (DFS), and the cut-off point for long-term oncological outcomes was 5-year. OS was defined as the date of diagnosis until death from any cause or the last effective follow-up, and DFS was defined as the date of diagnosis until death, recurrence, or the last effective follow-up.

### Statistical Methods

SPSS software (Version 22.0, SPSS Inc., Chicago, IL, USA) was used for statistical analysis, and the PSM extension of SPSS 22.0 software was used to achieve propensity score matching (PSM). Measurement data are expressed as the mean ± standard deviation, and an independent sample t-test was used for comparisons between groups. Count data are expressed as percentages (%), and the chi-square test was used to compare intergroup rates. Kaplan-Meier curves were drawn to analyze survival, and log-rank tests were used to compare differences in the survival curves. Multivariate Cox regression was used to analyze and determine independent risk factors, the relevant risks, and confidence intervals. In this study, P < 0.05 was considered statistically significant.

## Results

### Case Screening Results

**Figure 1:**
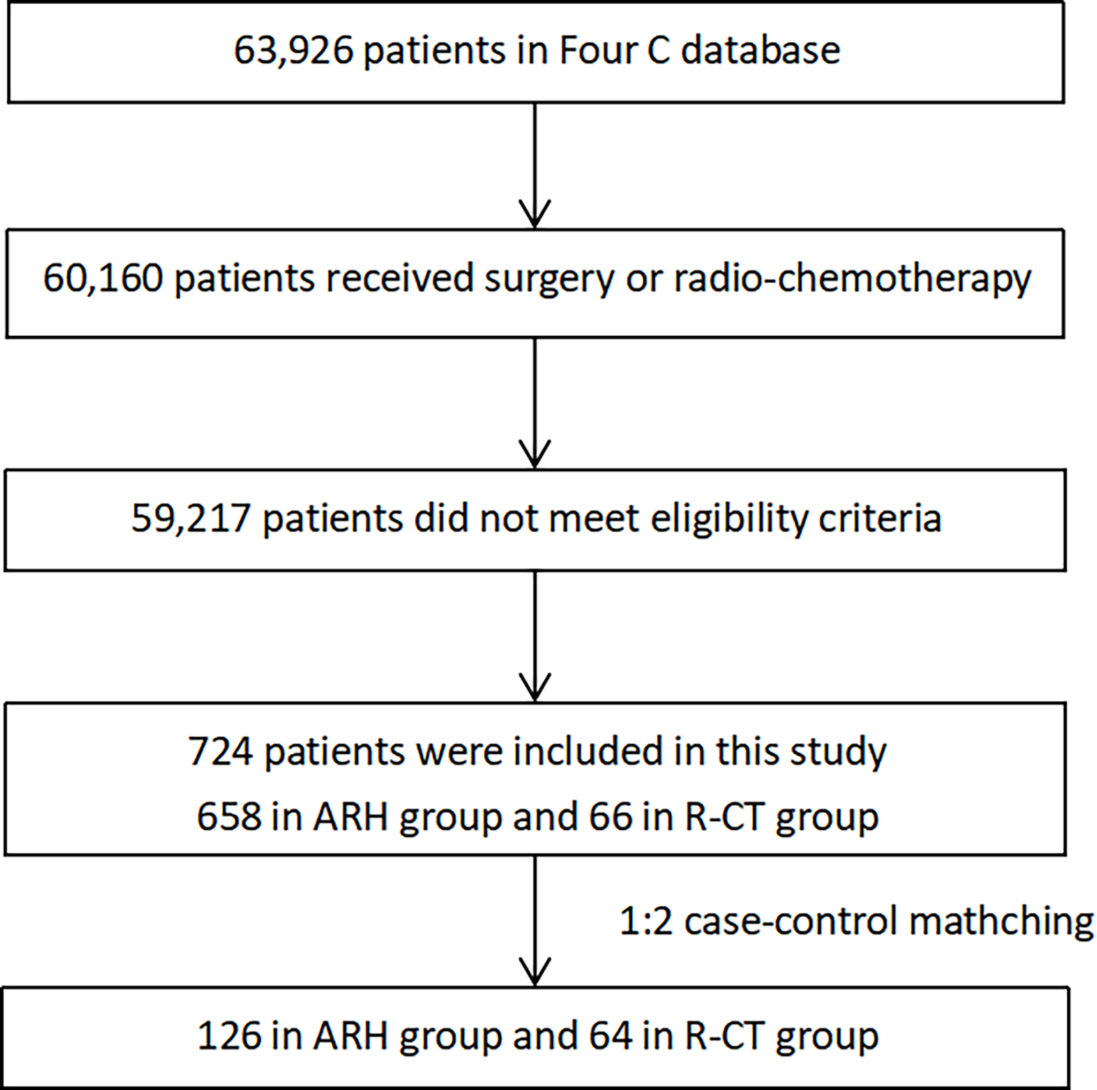
Flow diagram of patient recruitment and exclusions.

This study screened 724 patients who met the inclusion criteria. The data screening process is shown in **Figure 1**.

### Recurrence Patterns

The median follow-up was 58 months (1–152 months). Up to the last day of follow-up, 107patients developed neoplastic recurrence, and the recurrence rate was 14.8% for 5-year. 14.1% of the ARH group patients and 21.2% in the R-CT group developed neoplastic recurrence. Besides, The R-CT group had shorter recurrence 16.43 ± 12.21 months for the R-CT group and 21.30 ± 13.74 months for the ARH group) time compared to the ARH group. Local recurrence was seen in 26 cases. Distant recurrence was seen in 34 cases. The recurrence pattern of the remaining 47 cases was unknown (**Table 1**).

**Table 1:** Recurrence patterns of patients in the ARH and R-CT groups.

Characteristic	ARH group (n=658)	R-CT group (n=66)
Recurrence		
NO	565 (85.9%)	52 (78.8%)
YES	93 (14.1%)	14 (21.2%)
recurrence time	21.30 ± 13.74	16.43 ± 12.21
Patterns of recurrence		
Local recurrence	24 (25.8%)	2 (14.3%)
Distant metastases	32 (34.4%)	2 (14.3%)
Unknown	37 (39.8%)	10 (71.4%)

### Prognosis Comparison of the ARH Group and the R-CT Group Before and After Matching

**Figure 2:**
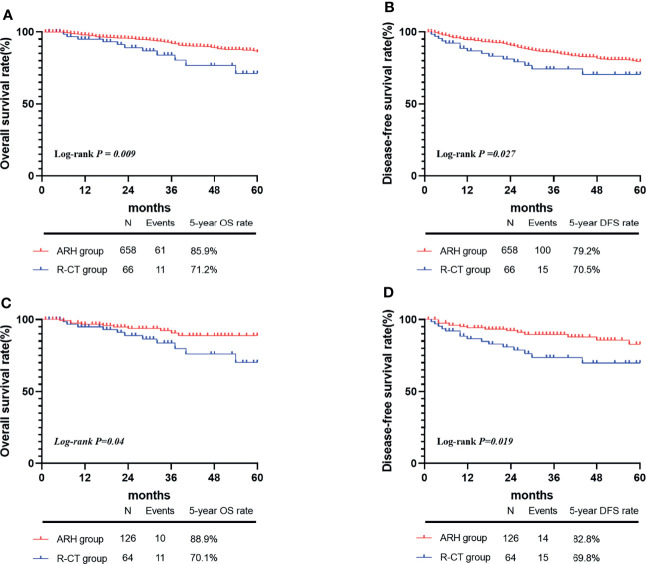
The 5-year OS and DFS rates of the ARH group and R-CT groups before and after 1:2 matching. Before matching, panels **A** and **B**; after matching, panels **C** and **D**.

A total of 724 patients met the inclusion criteria: 658 in the ARH group and 66 in the R-CT group (**Table 2**). Among the overall study population, Kaplan-Meier curve analysis showed that both the 5-year OS and DFS in the ARH group were significantly higher than those in the R-CT group (OS: 85.9% *vs.* 71.2%, *P*=0.009; DFS: 79.2%*vs.* 70.5%, *P*=0.027) (**Figures 2A, B**). Cox multivariate analysis showed a higher risk of death or recurrence in the R-CT group than in the ARH group (HR=3.19, 95% CI: 1.592-6.956, *P*=0.001) and worse DFS (HR=2.089, 95% CI: 1.194-3.656, *P*=0.01) (**Table 3**).

**Table 2:** Clinicopathologic characteristics of patients in the ARH and R-CT groups.

Characteristic	Before propensity score matching			After propensity score matching		
	ARH group (n=658)	R-CT group (n=66)	P value	ARH group (n=126)	R-CT group (n=64)	P value
**Age**	52.13 ± 9.74	58.11 ± 8.54	<0.001	57.67 ± 8.35	57.67 ± 8.30	0.997
**Tumor size**	2.97 ± 0.89	3.16 ± 0.98	0.094	3.04 ± 0.81	3.19 ± 0.97	0.249
**Histological type**			0.320			0.619
Squamous cell carcinoma	605 (91.9%)	64 (97%)		118 (93.7%)	62 (96.9%)	
Adenocarcinoma	36 (5.5%)	1(1.5%)		5 (4%)	1 (1.6%)	
Adenosquamous carcinoma	17 (2.6%)	1 (1.5%)		3 (2.4%)	1 (1.6%)	

ARH, abdominal radical hysterectomy; R-CT, radio-chemotherapy; OS, overall survival; DFS, disease-free survival.

**Table 3:** Cox multivariate survival analysis before propensity score matching.

Before propensity score matching	Multivariate analysis for 5-year OS			Multivariate analysis for 5-year DFS		
	P	HR	95% CI	P	HR	95% CI
**ARH *vs.* R-CT**	0.001	3.19	1.592-6.956	0.01	2.089	1.194-3.656
**Age**	0.025	0.97	0.944-0.996	0.134	0.984	0.963-1.005
**Tumor size**	0.478	0.918	0.724-1.164	0.478	0.918	0.724-1.164
**Histological type**	0.04			0.001		
Squamous cell carcinoma						
Adenocarcinoma		3.052	1.367-6.816		3.593	1.983-6.51
Adenosquamous carcinoma		1.928	0.598-6.215		1.306	0.412-4.142

ARH, abdominal radical hysterectomy; R-CT, radio-chemotherapy; OS, overall survival; DFS, disease-free survival; CI, confidence interval; HR, hazard ratio.

Due to differences in age between the ARH group and the R-CT group in the overall study population, 1:2 PSM was performed. After PSM, 126 patients were included in the ARH group, and 64 patients were included in the R-CT group **(** **Table 2**). These results were consistent with those of the whole study population. Kaplan-Meier curve analysis showed that both the 5-year OS and DFS in the ARH group were higher than those in the R-CT group (OS:88.9% *vs.* 70.1%, *P*=0.04; DFS:82.8% *vs.* 69.8%, *P*=0.019) (**Figures 2C, D**). Multivariate Cox analysis showed a higher risk of death or recurrence in the R-CT group than in the ARH group (HR=2.391, 95% CI: 1.051-5.633, *P*=0.046) and worse DFS (HR=2.6, 95% CI: 1.25-5.409, *P*=0.011) (**Table 4**).

**Table 4:** Cox multivariate survival analysis after propensity score matching.

After propensity score matching	Multivariate analysis for 5-year OS			Multivariate analysis for 5-year DFS		
	P	HR	95% CI	P	HR	95% CI
**ARH *vs.* R-CT**	0.046	2.391	1.051-5.633	0.011	2.6	1.25-5.409
**Age**	0.834	0.994	0.944-1.048	0.366	0.979	0.936-1.025
**Tumor size**	0.195	0.738	0.466-1.168	0.011	0.617	0.425-0.896
**Histological type**	0.982			0.513		
Squamous cell carcinoma						
Adenocarcinoma		2.426	1.582-6.049		2.364	0.549-10.176
Adenosquamous carcinoma		2.063	0.632-7.012		1.306	0.412-4.142

ARH, abdominal radical hysterectomy; R-CT, radio-chemotherapy; OS, overall survival; DFS, disease-free survival; CI, confidence interval; HR, hazard ratio.

## Discussion

According to the FIGO2018 staging system for cervical cancer, all patients with lymph node metastasis were classified as the newly established stage IIIC, and patients with stage IIA1 cervical cancer with lymph node metastasis were no longer included. However, the 2020 NCCN Guidelines for Cervical Cancer are consistent with the previous treatment principles of FIGO2009 stage IIA1 cervical cancer: (1) radical hysterectomy + pelvic lymphadenectomy (evidence level 1) ± para-aortic lymphadenectomy (evidence level 2B), and sentinel lymphadenography can be considered. (2) For patients with contraindications or refuse surgery, external pelvic irradiation + vaginal brachytherapy ± concurrent chemotherapy containing platinum is recommended (3). With changes in the principles of the FIGO2018 staging system, are the above treatments still suitable for patients with stage IIA1 cervical cancer? To answer this question, we conducted this multi-centre, large-sample retrospective study to compare long-term oncological outcomes in patients with FIGO2018 stage IIA1 cervical cancer who were cured by ARH and R-CT to obtain the optimal treatment strategy.

The oncological outcomes in the ARH group were better than those in the R-CT group in the overall study population. After controlling for confounding factors, such as age and histological type by 1:2 PSM, the 5-year OS and DFS of the ARH group were still higher than those of the R-CT group. In this study, the overall recurrence rate was 14.8%. The results are consistent with the recurrence rate of cervical cancer reported in previous studies (5%-40%). The R-CT group had a higher recurrence rate but shorter recurrence time compared to the ARH group, but there was no statistical difference between groups. Based on previous reports, we opted to combine cervical, vaginal vault, pelvic LN, and pelvic wall recurrences as LRR, and organ metastasis, peritoneal carcinomatosis and extra-pelvic LN recurrences as DR (9). Heterogeneity exists between different recurrence patterns in cervical cancer patients in previous studies (10–14). In our study, there was also statistical difference in recurrence modes between the two groups(*P*<0.05). In ARH group, the proportion of distant metastases (**34.4%**) was higher than that of local metastases (**25.8%**). Since 71.4% of the recurrence cases in the R-CT group had unknown metastasis sites, there were only 2 cases of local and distant metastasis respectively. However, additional information on the specific site of recurrence is needed for further research. This finding suggests that FIGO2018 stage IIA1 patients with cervical cancer may benefit from radical hysterectomy with standard postoperative therapy.

Previous articles comparing the oncological outcomes between ARH and R-CT in cervical cancer patients were based on the FIGO2009 staging system. In 2009, Bansal N (15) found that in stage IB1-IIA2 cervical cancer, the oncological outcome of patients with a tumor diameter < 6 cm receiving surgical treatment was better than that of the same patients receiving radiotherapy, and the study results of Rungruang B (16) were similar. In our study, 5-year OS and DFS were adopted as the observation outcome, so the results were more accurate. In addition, patients who received standard postoperative adjuvant treatment were included in this study, and the effects of non-standard postoperative treatment and other factors on the prognosis of patients were excluded. Moreover, the results and conclusions of this study were consistent with previous findings of our team (4).

However, findings from some studies are inconsistent with our findings. Landoni F (17, 18) found no difference between the survival outcomes of radiation therapy and surgical treatment in patients with stage IB1-IIA2 cervical cancer, possibly due to factors such as the small sample size in their studies, non-standard postoperative assisted treatment, and uncontrolled bias. The same problem was also present in the research of Yamashita H, et al. (19) In 2017, Wu SG et al. (20) analyzed 3,653 patients who received RH and 116 patients who received R-CT, and the results of PSM showed no significant difference in the effect of the two treatment methods on the prognosis of patients with stage IB1 and stage IIA1 cervical cancer, but the study also included patients with stage 1B cervical cancer. Furthermore, there was a large difference in the number of patients between the two groups, and the reliability of the PSM results was relatively low.

Compared with previous reports or articles on the optimal treatment strategy for cervical cancer, this study has certain advantages. On the one hand, there is currently a lack of relevant literature reports on the treatment strategy for FIGO2018 stage IIA1 cervical cancer, and previous studies were based on the optimal treatment plan for FIGO2009 stage IIA1 cervical cancer. However, patients with lymph node metastasis were not excluded from those studies. This study supplemented the relatively new research results in this aspect. On the other hand, this study was a multi-centre retrospective study that covered the case information of 1070 cervical cancer patients from 47 hospitals in various regions of China. At present, the current project represents a relatively comprehensive perspective of cervical cancer clinical diagnosis and treatment on a large number of patients worldwide. Due to the sufficient number of samples, we conducted multi-angle, multi-level, and multi-azimuth analyses of each stage of cervical cancer and adopted PSM to balance the baseline differences, which made the results more reliable.

We acknowledge several limitations to this study. This was a retrospective study, and there may have been confounding factors and biases, but these differences were balanced to a large extent through PSM; Although this study did not completely cover all regions in China and there are different medical levels in different regions, this database still represents the most comprehensive database on the diagnosis and treatment of cervical cancer patients in China.

In conclusion, our study found that for stage IIA1 cervical cancer patients, abdominal radical hysterectomy (ARH) with postoperative standard therapy offers better overall survival and disease-free survival outcomes than radio-chemotherapy (R-CT). This finding is consistent with the treatment recommended by the 2021 NCCN guidelines. Of course, the treatment strategy for FIGO2018 stage IIA1 cervical cancer still requires further prospective studies for verification.

## Data Availability Statement

The original contributions presented in the study are included in the article/supplementary material. Further inquiries can be directed to the corresponding authors.

## Ethics Statement

The studies involving human participants were reviewed and approved by Institutional Ethics Review Board of the Nanfang Hospital. Written informed consent for participation was not required for this study in accordance with the national legislation and the institutional requirements.

## Author Contributions

Conception and design: CC, PL, WL, and HD. Administrative support: CC and PL. Provision of study materials or patients: XZ, ED, QL, QX, RL, YX, JL, PL, and CC. Collection and assembly of data: XC, WL, HD, and MW. Data analysis and interpretation: XC, WL, HD, MW, and XB. Manuscript writing: All authors. All authors contributed to the article and approved the submitted version.

## Funding

This study received funding from the National Science and Technology Support Program of China (2 Grant No. 014BAI05B03), the National Natural Science Fund of Guangdong (Grant No. 2015A030311024), and the Science and Technology Plan of Guangzhou (Grant No. 158100075).

## Conflict of Interest

The authors declare that the research was conducted in the absence of any commercial or financial relationships that could be construed as a potential conflict of interest.

## Publisher’s Note

All claims expressed in this article are solely those of the authors and do not necessarily represent those of their affiliated organizations, or those of the publisher, the editors and the reviewers. Any product that may be evaluated in this article, or claim that may be made by its manufacturer, is not guaranteed or endorsed by the publisher.
